# Supply security beyond mines and scrap recycling: valorization potential of metallurgical residues

**DOI:** 10.1098/rsta.2023.0237

**Published:** 2024-11-04

**Authors:** Stefan Steinlechner, Kerrin Witt

**Affiliations:** ^1^ Department of Metallurgy, Chair of Nonferrous Metallurgy, Christian Doppler Laboratory for Selective Recovery of Minor Metals Using Innovative Process Concepts, Montanuniversität Leoben, Franz Josef-St. 18, Leoben 8700, Austria

**Keywords:** scarce metals, industrial residues, metallurgical by-products, extractive metallurgy

## Abstract

In the context of the European Critical Raw Materials Act, this work attempts to demonstrate the potential of residual material flows from non-ferrous metallurgy and their possible contribution to the supply security of metals by locally available new secondary resources, assuming technically and economically viable processing. Based on the aluminium, zinc, copper and lead industries, the resulting waste streams are discussed and, in particular, the complex process consisting of physical, chemical and metallurgical steps is described. Their diversity, be it slags, dusts or even sludges, has a wide variety of morphologies and compositions due to the process of generation. In the past, many concepts for reprocessing were investigated, but the goal was usually only the recovery of one target element or to avoid landfilling by using it, for example, as a building material, whereby the metals contained are completely lost. If the target is the extraction of valuables, the required interdisciplinary process development must be based on an in-depth characterization to understand the behaviour of metals and trace elements in possible extraction steps and also to develop suitable strategies for influencing the behaviour of target elements with the aim of extraction. This starts with an in-depth comprehension of the formation process, which is the subject of this article and has a direct influence on the composition and morphology of the materials, thus forming the basis for understanding the behaviour in potential recycling processes. Furthermore, typical compositions of the residual material streams, sources and, if available, quantities are shown and, in summary, an attempt is made to evaluate the materials in a SWOT analysis and to address the challenges in developing extraction steps for processing. While mine tailings are mostly found outside of Europe, the potential of the residual materials from metallurgy is local due to the processing of the concentrates in Europe. This leads to several potential advantages in a possible reprocessing, such as no or shorter transport routes, which is linked to lower quantity of emissions, defined volume and known composition, no geopolitical risk, conservation of primary resources, and increasing Europe’s sustainability through a more comprehensive use of the raw materials.

This article is part of the discussion meeting issue ‘Sustainable metals: science and systems’.

## 1. Introduction

Metals, owing to their uses in infrastructure, mobility, energy supply or high technology, play an integral role in the modern world. Trends such as the transition to a low-carbon economy, including renewable energy and electrification of transport, are driving the metal consumption—base metals as well as speciality metals—in Europe [1]. With its ‘Critical Raw Materials Act’, the European Union is setting targets for a more independent supply of raw materials in Europe, including a stronger focus on recycling and at the same time enhancing local primary extraction and processing [2,3]. However, the gap between demand and supply cannot be covered in the short term solely by the development of new mines, which is the first main pillar to be addressed in Europe. The second pillar that contributes to the security of raw material supplies, alongside responsible mining, namely the recycling of selected metals from old products, will not be able to close this gap in demand alone as long as demand does not stagnate. This is why a combined approach is needed that can include the use of unused industrial by-products and residual materials as a possible third pillar, for example, next to sustainable use and production. In this context, this publication examines the potential of generated residues from the metallurgical base metals industry to contribute to a better supply security of selected metals within the European Union in the case of a reprocessing, starting from the base metals themselves but continuing throughout selected hitch-hiker metals which can be present in those process streams from the base metals production. It is well known in the metallurgical industry, for instance, illustrated by the metal wheel [4], that many of the technologically important metals are obtained exclusively as by-products of the base metals industry and therefore have no primary production of their own. This is why the material flows of the base metals industry and the corresponding residues and by-products are so interesting, as they are not only a potential source for the base metals such as aluminium, zinc, lead, copper and nickel, but also a possible source for many of the accompanying hitch-hikers, such as cobalt, indium, precious metals and others. A thorough understanding of the materials under consideration is necessary to assess potential uses for by-products or to create independent treatment concepts, as is frequently required. As various residues along the raw material processing chain are generated, this article will focus on the potential of residues from non-ferrous metallurgical industry and within this to the base metal commodities copper, zinc, lead and aluminium, responsible for the highest amount of residues and potentially carrying a high number of technologically interesting hitch-hiker metals. The residues that are the subject of this discussion are not tailings, but rather materials that have undergone significant physical, chemical and metallurgical transformations, such as slag, dust, sludge, precipitation products or also auxiliary materials such as spent refractory bricks, that should be taken into account during the process development [5,6].

## 2. Background

A large percentage of the metals demand in Europe are either imported as a metal, metal compound or in the form of finished goods, such as in the case of aluminium, rare earth elements, cobalt or nickel, which are crucial for enabling the production of products for instance for the green energy transition. A large proportion of these important technology metals, such as rare earth elements (REE), indium, etc. are mostly hidden in imported products, like permanent magnets, electronics, displays or photovoltaic (PV) modules. A major reason for this is the strong dominance of some producing countries, such as China, Russia, Japan, Taiwan or America, to name just a few outside of Europe [7,8]. If Europe does not receive semi-finished goods or products, it will import the already-produced metals themselves or alternatively the primary ore concentrates that need to be processed to extract the corresponding metals inside Europe, which again creates a strong dependence on concentrate-supplying countries. In some of the metal production cases, the complete raw material processing chain, and a significant share of consumed metal, can be found in Europe, starting with mining, mineral beneficiation up to the hydro- or pyrometallurgical extraction of metal from the ore concentrates. In general, it can be stated that all raw materials play an essential role in the stability of Europe’s economy and the production landscape, regardless of whether talking about technology metals, usually produced in small quantities and as a by-product of base metals, or about the base metals themselves. For this reason, it is important not only to conclude strategic international raw material partnerships but also to develop and rely on locally available resources against the background of securing raw material supply for Europe. This last aspect can either be realized by opening new primary mines, increase of recycling rates or by developing new secondary resources.

The development of a new primary mine project typically requires several years, starting with prospecting, project planning, evaluation by different competent persons using standards such as 43 101 or Joint Ore Reserves Committee codes, financing and ramping up, all assuming that the necessary permits and environmental assessments are available. Given this background, primary mines are a necessary long-term strategy, but unless the projects are already in development, they are not a solution to a short-term metal shortage [5,9–11].

Theoretically, increasing recycling rates can help bridge short-term supply bottlenecks. An important aspect in this context, however, is that the recovery rates in metallurgical recycling processes are already very high and a significant increase can usually only be achieved in upstream process steps such as collection or beneficiation. A generally higher proportion of recycled content in total metal production of specific metals, such as aluminium, is also limited due to the constantly increasing materials demand/production today and the parallel limited return rate of scrap due to typical product lifespans of several years. On the other hand, this means that in the future, when demand approaches plateau, a significant part of the supply might be attributable to recycling [4].

The third mentioned field is the development of new secondary resources, focusing on by-products and residues from metal production, which are generated along the natural resource processing chain. By tracing the natural path of the ore in mind, the following by-products/residues can be theoretically identified: topsoil, mining waste (overburden, waste rock, spoils and mine water), processing waste from mineral beneficiation (tailings from flotation or other processes, wastewater), metallurgical waste (flue dust, precipitates, sludge, slag, ash, process water and spent refractory material), or also production waste (dross, chips, etc.) [5].

The topsoil and mine wastes are mostly non-economic materials and do not carry significant content of valuable metals. This does not mean that they cannot be utilized for instance as refilling material for mines, but they are not considered a new source for metal extraction. On the other hand, processing wastes from mineral beneficiation, a enrichment step after mining, can be a possible new source. However, the material was at the time of the excavation and processing below the economic cut-off grade of the ore, which is the reason for its declaration as residue. To become economically viable, this cut-off grade would have to change over time or a not recovered accompanying element move into focus. Examples of this are copper concentrates, which get reprocessed for copper, gold or cobalt, or also the extraction of tantalum and niobium from tin tailings. However, this is rather the exception. The greatest potential, though often overlooked, for new sources of raw materials lies in the area of metallurgical by-products and residues. Since industrial plants are typically built for decades of operation and some of the metals in focus today gained their relevance recently, plants are often lacking infrastructure for extraction. This means that various valuable elements are separated from the process stream of the main commodity metal via the by-products/residues from metallurgy, which are still dumped in many cases [5].

An additional challenge for the metallurgical industry is the tendency towards deteriorating quality of mined ores, accompanied by a continuous decrease of the cut-off grade over the last decades. Very often the poor quality of resource must be compensated by a higher extracted ore volume, which leads to higher effort towards beneficiation, especially grinding and linked energy consumption, and leading to an increase in processing costs per tonne of shipped ore concentrate. This circumstance means that residues that previously seemed uninteresting are now becoming the focus of processing efforts. Another aspect in this context is the fact that more and more polymetallic ores are available in the market, increasing the input of element diversity into the existing plants which in a lot of cases still end up in the corresponding residues, due to a lack of extraction infrastructure. Still, the increasingly stringent environmental protection and landfill legislation, together with decreasing landfill space, have raised monitoring costs, resulting in higher efforts to produce in a resource-efficient way and, thus, to extract even those hitch-hiker metals from industrial intermediate or waste streams.

By utilizing these waste streams, Europe could not only uphold the principle of resource conservation but also significantly improve the efficiency with which imported raw materials are used, thereby increasing resource efficiency and decreasing its associated environmental impacts.

## 3. By-products and residues from non-ferrous metallurgy

To evaluate possible areas of utilization of by-products or to develop independent treatment concepts, as is often necessary, a comprehensive understanding of the materials in focus is required. In contrast to tailings, the residues focused on here are materials that do not correspond to the structure and morphology of the processed ore, but rather represent a material that has undergone physical, chemical and metallurgical treatments and with these significant transformations to be considered in the process development [12,13].

Although not all metals and accompanying metals (hitch-hiker metals) can be discussed, an important detail should be mentioned here, namely the fact that many technologically important metals do not have primary ores and are extracted exclusively or at least to a great extent as by-products of the base metals. This affinity of individual metal compounds to each other does not only exist in nature; this geological peculiarity also extends from the geological deposit (mine) through the preparatory beneficiation processes for enrichment, to the metallurgical steps that attempt to separate these mineralogical associations and produce individual metals. For this reason, not only are the base metals themselves found in the waste products of the corresponding industry, but potentially also all other accompanied hitch-hiker metals in the processed ore concentrates, which is also illustrated in the metals wheel [4]. Table 1 gives a brief insight into selected, currently highly focused hitch-hiker metals, their origin, typical extraction efficiency, and estimated contribution to the economy of the relevant base metal industry. Although not all of the hitch-hiker metals mentioned in the table as examples are discussed in this article, it underlines the importance of the primary industry (aluminium, zinc, lead, copper and nickel) and, as described later, their by-products/residues as a potential source of those metals. Rare earth elements, which according to the table are considered only a by-product of the iron industry, next to their occurrence in primary ores, are also found in residues from non-ferrous metallurgy and are therefore mentioned here. In this context, the table shows the frequent but not exclusively geological association of REE with iron compounds and their possible extraction to a certain extent from different iron-containing materials. Such mentioned materials include slags from iron production but also other iron-containing carrier ores, such as those from zinc or aluminium production. This explains why small amounts of REE can also be found in iron-containing residues from non-ferrous metallurgy, such as red mud from the aluminium industry, or in iron precipitation residues from the zinc industry [15–17]. The last column is an important factor because it decides how willing the industry is to build new infrastructure for its extraction or change the production quantity of the base metal and thus the supply side of the market leading to price volatility, to counteract shortages of hitch-hiker metals.

**Table 1.** Selected examples of hitch-hiker metals from primary base metals production (zinc, lead, copper, nickel, aluminium and iron), grouped according to the production source representing the carrier-metal concentrate, the production share representing the proportional contribution to the total by-product supply (from all sources), the recovery efficiency represents the typical extraction rate in the course of the mentioned carrier-metal production, and the last column outlines the estimated contribution to the total revenue of the carrier-metal plant [14].

hitch-hiker metal	sources of production	share of production	recovery efficiencies	maximum share of total revenues
cobalt	nickel	55%	75–90%	~15%
	copper	35%		~15%
	primary	10%	—	—
gallium	alumina	90%	10%	~4%
	zinc	10%	—	—
germanium	zinc	70%	~12%	~2%
	coal	25%	—	—
gold	primary	~90%	—	—
	copper	~10%	>99 %	~20 %
indium	zinc	~100%	25–30%	~3%
palladium	platinum	60%	40–60%	~15%
	nickel	40%	—	~15%
platinum	nickel	15%	—	~10%
rare earths	iron	45%	—	—
	primary	55%	—	—
rhenium	copper	100%	~75%	~0.3%
silver	lead-zinc	35%	>95%	~45%
	primary	30%	—	—
	copper	23%	>99%	~25%
	gold	12%	—	—

Looking at the table, it is obvious that even if there is an infrastructure for the extraction of accompanying metals, the low extraction efficiency in different cases leads to a certain content in the industrial wastes. Be that as it may, many industries do not recover all these hitch-hiker metals anyway, which in turn leads to their accumulation in the waste streams. Although these materials contain valuable metals, many of these residues/by-products are still in stockpiles due to their complex morphology and structure, which means that reprocessing must take a comprehensive approach and begin with a deep understanding of the characteristics of the material, which are directly influencing the behaviour in possible metallurgical processing steps and provide the basic knowledge to develop steps to influence behaviour [9,18].

Although it is difficult within the scope of this article to show in detail where exactly the respective hitch-hiker metals remain and which processes they go through, a basic understanding of their processes of origin (base metals industry) and thus the physical, chemical and metallurgical transformation that influence the material is essential for any further research. In addition to the base metals industry, there are also other interesting and potential sectors with their resulting residues, such as ferroalloy production, which, however, are not discussed here [19].

### (a) Aluminium industry

**Figure 1.**
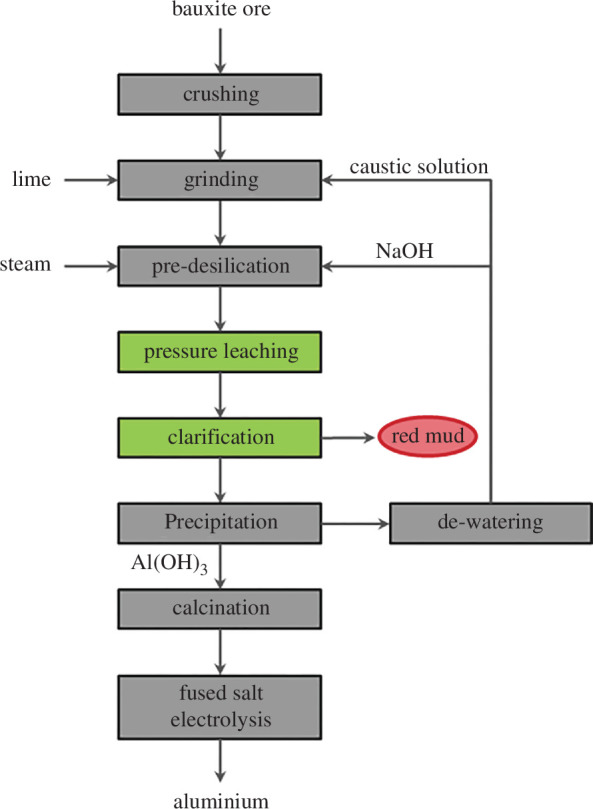
Flowsheet of aluminium oxide production [20].

The most frequently produced non-ferrous metal in terms of volume today is aluminium. The state-of-the-art production is split into a red part, the so-called Bayer process, and a white part, the oxide production, which is then further utilized as input material in the Hall–Héroult fused salt electrolysis to produce metallic aluminium. Figure 1 outlines the required steps to produce a pure Al_2_O_3_ calcine material. Today, bauxite is usually used as a collective term for all hydroxide alumina minerals. Bauxites are composed of hydroxides and oxyhydroxides of aluminium and contain SiO_2_, TiO_2_ Fe_2_O_3_, V_2_O_5_, Cr, P, F, various other minor elements such as Ga, Sc and organic substances as admixtures.

Based on the bauxite quality, it can be roughly assumed that 4 tonnes of bauxite are processed into 2 tonnes of Al_2_O_3_ and these into 1 tonne of metallic Al. The initial step of the bauxite ore involves crushing and a wet grinding. This is carried out at a temperature of approximately 60°C and involves the addition of spent Bayer liquor, an alkaline NaOH solution. A portion of the aluminium hydroxides and silicate minerals, such as kaolinite, start to dissolve, as shown in following equation:


$$3Al_{2}Si_{2}O_{3}{\left(OH\right)}_{4}+18NaOH↔6Na_{2}SiO_{3}+6NaAl{\left(OH\right)}_{4}+3H_{2}O.$$

During the alkaline digestion of siliceous bauxites in the autoclave, sodium aluminium silicates are formed which, together with red mud particles (the main residue from aluminium industry in terms of quantity), form dense crusts on the heating surfaces of the preheaters and in the autoclave and thus impair heat transfer. That is why many alumina factories today carry out a pre-desilication after a wet grinding. Therefore, the bauxite suspension is heated to approximately 90°C in large containers and left to stand for 6–10 hours while stirring, which forms the insoluble hydroxide sodalite (3Na_2_O⋅3Al_2_O_3_⋅6SiO_2_⋅2NaOH) and avoids its formation in a later stage in the autoclaves. The corresponding equation can be mentioned as follows:


$$6Na_{2}SiO_{3}+6NaAl{\left(OH\right)}_{4}+Na_{2}X↔Na_{6}\left[Al_{6}Si_{6}O_{24}\right]*Na_{2}X+12NaOH+6H_{2}O.$$

The formed so-called Bayer sodalite can incorporate at the X position a range of inorganic anions, such as carbonate, hydroxide, sulfate or also chloride if present. It cannot be separated from the rest of the slurry and remains during the process, until the separation of the red mud, and is one of the reasons why the red mud always carries a significant percentage of aluminium as well.

The digestion itself is carried out in a pressure leaching step at temperatures of 250–300°C under the addition of concentrated sodium hydroxide, following the below chemical reactions:


$$Al{\left(OH\right)}_{3}+NaOH↔Na^{+}+Al{\left(OH\right)}_{4}^{-}.$$

$$AlO\left(OH\right)+NaOH+H_{2}O↔Na^{+}+Al{\left(OH\right)}_{4}^{-}.$$

As a result, a saturated Al(OH)_4_^–^ solution still carrying insoluble particles is obtained. The conditions in the pressure leaching allow an almost complete dissolution of aluminium compounds, excluding the previously formed Bayer sodalite. The pressurized solution is then relaxed, and the solid particles are removed within a clarification process, separating the so-called red mud residue at approximately 2 tonnes per 1 tonne of produced metallic aluminium. As a support, flocculant chemicals are added in the thickener. Table 2 summarizes compositions of different red mud residues from industrial production of different bauxite materials. Based on literature, different scarce elements can be found in the red mud as well, introduced by the bauxite raw material. Examples of scarce metals are scandium in the form of Sc_2_O_3_ (<0.014 wt% [25]), ZrO_2_ (<0.147 wt% [25,26]), V (<730 ppm [27,28]) and different rare earth elements in quantities up to several ppm [27,29,30].

**Table 2.** Chemical composition (main compounds) of dried red mud from different bauxite sources.

wt%	Fe_2_O_3_	Al_2_O_3_	CaO	TiO_2_	Na_2_O	SiO_2_
Greece [21]	44.6	23.6	11.2	5.7	2.5	10.2
India [22]	35−54	17−22	1−5	3−19	3−6	4−16
Australia [23]	28.5−56.9	15.6−23.2	2.25−5.26	2.65−8.03	2.2−8.6	3−30
Brazil [23]	45.6	15.1	1.16	4.29	7.5	15.6
Germany [23]	44.8	16.2	5.22	12.33	4.0	5.4
Spain [23]	37.5	21.2	5.51	11.45	3.6	4.4
USA [23]	35.5	18.4	7.73	6.31	6.1	8.5
Jamaica [24]	42.3−49.5	16.4−16.5	5.5−9.1	6.0−7.0	2.3−4.6	3.0−8.0

The relaxed and cooled-down solution, already separated from the red mud, represents a supersaturated Al(OH)_4_^–^ solution, which requires a seeding process to start the kinetically inhibited crystallization of aluminium hydroxide [23,31,32]. This is achieved by returning species-specific seeds from the following step.


$$Na^{+}+Al{\left(OH\right)}_{4}^{-}↔Al{\left(OH\right)}_{3}+NaOH.$$

After separating and washing the formed Al(OH)_3_ it is partly utilized as a seeding agent while the remaining amount is further processed in a calcination step for hydroxy group separation. At a temperature of approximately 960°C it is transformed to Al_2_O_3_ following the reaction below:


$$2Al{\left(OH\right)}_{3}↔3H_{2}O+Al_{2}O_{3}.$$

This high-purity alumina is used as a feedstock for Hall−Héroult fused salt electrolysis for the production of metallic aluminium. A more detailed description of the electrolysis process is omitted at this point. However, worth mentioning in the context of this article is the spent refractory material from the Hall−Héroult cell, which nowadays is mainly sent to a waste landfill and is described in §3*e*. In the context of the recovery of accompanying elements from these spent refractories, a special kind of residue from aluminium industry, is of only minor importance. Additionally the generated amount of spent refractory per tonne of produced aluminium is relatively low. One reason for the low possible impact of these material is the long service life of the refractory bricks in the Hall−Héroult fused salt electrolysis cells and on the other side , since the separation of of impurities and accompanying elements already takes place before electrolysis in the Bayer process, via the red mud, only minor amount of valuable trace elements can be expected.

### (b) Copper industry

**Figure 2.**
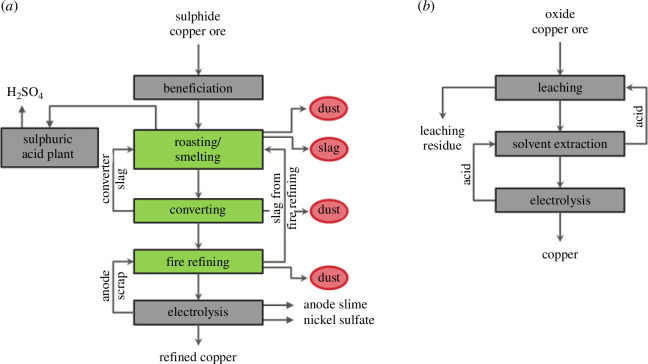
(*a*) Flowsheet of the pyrometallurgical processing steps for copper production. (*b*) Typical hydrometallurgical copper extraction [35].

Copper, with its high electrical and thermal conductivity, plays a crucial role in Europe’s energy transition [33]. This, and its additional, versatile properties make it the non-ferrous metal with the second highest demand after aluminium [34]. About 80% of the primary copper originates from sulfide ores containing minerals like chalcopyrite (CuFeS_2_) or bornite (Cu_5_FeS_4_). Average concentrations of these minerals in typically mined ores are low, containing around 0.5% Cu (open pits), or 1–2% Cu (underground mines). The extraction of copper from these ores after isolating the Cu minerals by flotation is realized by pyrometallurgical processes. Oxide ores, on the other hand, contribute the remaining 20% of the total copper mine production, and are processed by hydrometallurgical methods [35,36]. Flowsheets of the respective principal processes are shown in figure 2.

In the hydrometallurgical route, copper is extracted from mainly low-grade oxide ores and also some sulfide ores, through leaching followed by purification to remove impurities using processes such as precipitation or solvent extraction, and lastly the electrowinning of pure cathode copper [35]. Due to the easy containment of liquid streams in the solvent extraction/electrowinning circuit and the fact that impurities are returned to the site where they originated, few opportunities for residue processing arise [37]. Therefore, in the following, this copper extraction route will not be investigated in more detail, given the purpose of this publication.

The pyrometallurgical processing of copper-iron sulfide concentrates involves several processing steps, including roasting/smelting, converting, fire refining and electrorefining. In the smelting process, sometimes preceded by a roasting step, the concentrate with around 30% copper is melted with added silica while the contained S and Fe are partly oxidized. This results in a Cu-enriched molten sulfide phase (matte, 50–70% Cu), a molten oxide slag as free from entrained or dissolved copper as possible, along with an SO_2_-bearing offgas [35]. The formation reactions are given in the following equations:


$$2\;CuFeS_{2}+\;\frac{13}{4}\;O_{2}↔\;Cu_{2}S\cdot \frac{1}{2}FeS+\frac{3}{2}\;FeO+\frac{5}{2}\;SO_{2},$$

$$2\;FeO+SiO_{2}↔\;Fe_{2}SiO_{4}.$$

The volatilized material is typically condensed and then reintroduced into the furnace, ensuring that all impurities ultimately leave the furnace in the form of either matte or slag. The offgas is then treated in sulfuric acid plants for SO_2_ removal while the state-of-the-art process for the generated slags consists of different treatments to recover the contained copper. The remaining inert slag can be used in various construction applications [38]. The molten matte is then transferred to a converter. Here, the so-called blister copper (98.5–99.5% Cu) is produced by blowing air or oxygen-enriched air into the matte in an autothermal process. The converting consists of two sequential stages: the first step is the oxidation of FeS from the molten matte and the formation of slag by addition of silica flux:


$$2\;FeS+\;3\;O_{2}+SiO_{2}↔\;Fe_{2}SiO_{4}+2\;SO_{2}.$$

The slag-forming stage is considered complete when the Fe content in the matte has been reduced to approximately 1%, so that most of the Fe can be removed as slag before the copper production. The respective slag is recycled into the preceding smelting process [39]. In the second step, copper is formed from the obtained impure Cu_2_S matte by oxidation of sulfur:


$$Cu_{2}S+\;O_{2}↔\;2\;Cu+SO_{2}.$$

The SO_2_-bearing offgas and the slag from the converting step are treated the same way as those originating from smelting; the offgas is sent for cooling, dust removal and H_2_SO_4_ production, the slag for copper recovery treatment [35,38]. The copper produced by smelting and converting effectively always undergoes electrorefining. For this, it is necessary to cast strong and smooth anodes that can be installed in electrolytic cells. The blister copper from the converting step still contains considerable amounts of S and O, which would react to gaseous SO_2_ during the solidification of newly cast anodes, resulting in bubbles and subsequently in weak anodes with a bumpy surface. Therefore, the molten blister copper needs an intermediate fire-refining step consisting of sulfur removal by air oxidation and subsequent oxygen removal by hydrocarbon reduction [35]. The slags generated in this pyrometallurgical refining step are normally recirculated to a prior process stage [38]. The metal is then suitable for casting anodes that enter the electrorefining process. Here, the copper from the impure anodes is dissolved into a CuSO_4_–H_2_SO_4_–H_2_O electrolyte, followed by selectively electroplating it onto cathodes without the anode impurities. Impurities like As, Bi, Co, Fe, Ni, S and Sb dissolve in the electrolyte, but remain there while Cu is plating, whereas other metal impurities (Ag, Au, platinum group metals, Se, Te, Pb and Sn) join the solid slimes, a valuable resource of precious metals and other scarce elements, that is subsequently treated for metal recovery. To prevent the soluble anode impurities from building up in the electrolyte, they are continuously removed from a bleed stream, recovering inter alia Ni sulfate [35].

Another major source for copper besides its extraction from primary raw materials is the recycling of copper and copper-alloy scrap. It can be processed either together with concentrates in a primary smelter/refinery or in specialized secondary copper plants, which combine similar procedures, i.e. smelting, converting, fire-refining and electrorefining. Depending on the scrap grade, the secondary material is added at different points of the processing chain. As the composition of the input materials differs from the primary concentrate, so do the formed dusts and slags. Metallic impurities like Fe, Ni, Pb, Sn or Zn enter the processes with the copper scrap and end up in different streams. Owing to their comparably high copper contents, the generated slags from converting and fire-refining are recycled to the smelting furnace. The oxidation in the converting step generates an offgas containing the volatilized metal oxides PbO, SnO and ZnO. They are commonly recovered from the dusts by reduction. In electrorefining, the electrolyte and the anode slime are treated equivalently to the primary route described earlier to extract the precious metals and nickel sulfate [35].

Copper metallurgy in general does not produce many residues that are not reprocessed for the extraction of the various scarce and valuable metals they contain. Many of them recirculate within the processing chain, minimizing the number of waste stream outlets. A residual material often overlooked is the spent refractory material from the various furnaces, which is currently landfilled to a great extent. This is described in more detail separately in §3e.

### (c) Zinc industry

The main mineral processed in the modern zinc industry is ZnS, also called sphalerite. Typical grades of mined ores are at 4% zinc content or above and undergo a typical beneficiation route for sulfidic ores, including crushing, grinding, flotation and dewatering. Depending on the deposit, pure zinc concentrates, or mixed lead–zinc concentrates, are produced. The former are processed by hydrometallurgical methods, while the latter are used as feed in pyrometallurgical plants. As mixed lead–zinc concentrates are declining, the pyrometallurgical route has become rare today. They are briefly discussed in the following §3d about lead industry. This section describes the hydrometallurgical processing of common pure zinc concentrates, which contain 45–60% Zn, 25–35 % S, 1–5% Pb, up to 12% Fe and traces of various other accompanying elements, such as Cu, Sb, As, Sn, Ag, Bi or Au [40,41]. Zinc production processes (whether hydrometallurgical or pyrometallurgical) require oxide feedstock. The conversion from sulfidic to oxidic material is realized by a roasting step. The commonly valid reaction for this is shown below:


$$ZnS+\;\frac{3}{2}O_{2}↔ZnO+SO_{2}.$$

During the fluidized bed roasting process other sulfides, such as FeS or PbS, are converted into oxides as well. A common side product from non-ferrous plants treating sulfidic ore concentrates, typically sold to the chemical industry, is sulfuric acid, produced from the SO_2_/SO_3_ containing offgas from the roasting step. The formation of spinel-type structures between ZnO and other oxides during the roasting are unwanted side reactions, due to the higher effort required in the leaching steps.


$$ZnO+Fe_{2}O_{3}↔ZnFe_{2}O_{4}.$$

$$2ZnO+SiO_{2}↔Zn_{2}SiO_{4}.$$

**Figure 3.**
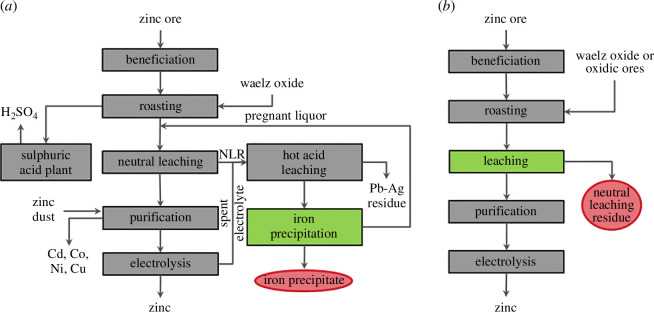
(*a*) Flowsheet of the commonly applied roast leach electrowinning (RLE) zinc winning via a two-step leaching. (*b*) RLE process for zinc production in case of low iron-containing ore concentrates [42].

The leaching of the roasted ore concentrates, also called calcine, is split into a neutral leaching (50–70°C, pH~5; 10–15 g/l H_2_SO_4_) and a hot-acid-leaching step (80–95°C, 120–140 g/l H_2_SO_4_). The main reason for this is the zinc ferrite mentioned above, which requires higher temperatures and higher acid concentration compared with the mineral ZnO to be dissolved. For economic reasons, the amount of solution for this more expensive step is to be kept as low as possible, which also means that the volume flow for iron precipitation can be kept smaller. While spent electrolyte from electrolysis is used for hot-acid-leaching due to its low pH value, the solution from solid/liquid separation after iron precipitation is used for neutral leaching. Typically, acid losses that occur are covered by the sulfuric acid produced at the site from the roasting gases. A valuable side product is the so-called Pb–Ag residue, which is the remaining solid part from the hot acid leaching, also carrying most of the SiO_2_, and is marketed as a product for the lead industry. The flow sheet of such a two-step leaching plant is illustrated on the left in figure 3, while the right flowsheet shows an adapted processing scheme for the case of low iron-containing concentrate. In this case, a separate step can be omitted due to the lower zinc losses caused by zinc ferrite. Due to the fact that ore concentrates tend to show lower zinc concentrations and associated higher iron contents, the two-stage leaching process is likely to become even more important in the future.

Due to the high content of dissolved iron, iron removal takes place in a separate step to the additional necessary solution cleaning (purification step), which will be discussed later. The main reason is the interference in the electrolysis cell and the cause to remove it to avoid impurities in the zinc metal product. Depending on various circumstances, such as a possible implemented indium recovery, either a jarosite or a goethite precipitation are the common precipitation methods, in addition to hematite precipitation, which is almost never used due to the costs.

The main difference between the goethite and jarosite process is the valence of the iron at the start of the precipitation process, resulting in a different mineral structure of the product. The resulting precipitate originating from one of the two mentioned iron precipitation processes above is named after the naturally occurring minerals jarosite (XFe_3_(SO_4_)_2_(OH)_6_) or goethite (FeO(OH)), having the same mineral structure as the produced precipitation product. For the precipitation in the form of jarosite, the divalent iron is oxidized to trivalent iron by the addition of calcine and/or MnO_2_. High temperatures of about 95°C and low acidity are required, assured by further addition of calcine, which also neutralizes the three moles of sulfuric acid formed during the jarosite precipitation. This causes losses of zinc, lead and silver, due to the calcine used for pH adjustment. The following equation outlines the general reaction of the formation of ammonium jarosite:


$$Fe_{2}{\left(SO_{4}\right)}_{3}+\frac{1}{3}{\left(NH_{4}\right)}_{2}SO_{4}+6H_{2}O↔\frac{2}{3}{\left(NH_{4}\right)}_{2}Fe_{6}{\left(OH\right)}_{12}{\left(SO_{4}\right)}_{4}+3H_{2}SO_{4}.$$

Today, European zinc plants are mainly producing sodium jarosite, while the ammonium jarosite still can be found on different dump sites. The jarosite is a basic iron sulfate with the general structure of R_2_Fe_6_(OH)_12_(SO_4_)_4_. R can be NH_4_^+^ but also other cations such as Na^+^, K^+^, or one-half of Pb^2+^ as well as Ag^+^, just to name a few. On the other side, the trivalent iron can be substituted by Th, Ga or also In, outlining the complexity of these precipitation residues [6,43–45].

As an alternative precipitation, the goethite process is commonly used. While above a certain level of iron (12 g l^−1^) in the solution the hydronium jarosite structure is stable, below a content of 2 g l^−1^ the goethite becomes more stable. For this reason, the typical iron concentration in the solutions is too high and needs to be diluted before precipitation occurs. In addition, a reduction of the trivalent to divalent iron is performed, utilizing sulfidic ore concentrate, producing a residue containing elemental sulfur, which is returned to the roasting step or landfilled


$$2Fe^{3+}+ZnS↔2Fe^{2+}+\;Zn^{2+}+S^{0}.$$

Afterwards, oxygen is used to oxidize the divalent iron in the solution to ferric iron and in parallel precipitate as a goethite mineral. In the same way as with jarosite precipitation, the goethite reaction causes the formation of 2 moles of sulfuric acid, which needs to be neutralized by calcine as well


$$2Fe^{2+}+2SO_{4}^{2-}+\frac{1}{2}O_{2}+H_{2}O↔2FeOOH+2H_{2}SO_{4}.$$

It can be seen in table 3 that the content of iron in goethite is higher than in jarosite; also the quantity to be landfilled is lower but at the same time, the loss of zinc is higher.

**Table 3.** Average compositions of iron precipitation sludges of different processes from the zinc industry (per 100 t of feed concentrate) [42].

process	jarosite	Vieille Montagne (VM) goethite	paragoethite	hematite
composition				
Fe (%)	29	40	34	57
Zn (%)	3.5	8.5	13	1
Pb (%)	1.9	1.9	2.2	0
quantity (t)	22.5	16.2	19.2	11.2

Some locations stabilize the jarosite with cement and lime before it is landfilled (Jarofix process), which on the one hand enables a safe disposal, but on the other hand results in increased land use and significant losses of valuable metals as it makes possible reprocessing more difficult. A comprehensive summary of the concepts examined for processing iron precipitates but also stabilized jarosite can be found in the paper by Höber and Steinlechner [12].

Before the pregnant solution can be used for the electrowinning of zinc, it has to undergo further purification. Impurities that are still contained in the solution can contaminate the cathode zinc, reduce the current efficiency and cause faults in the cell operation. Arsenic, antimony, selenium, tellurium and germanium influence the hydrogen overvoltage in electrolysis. Lead, cadmium and tin contaminate the cathode zinc through co-deposition and aluminium, while manganese and magnesium lead to an increase in the viscosity of the electrolyte. Furthermore, nickel, copper and cobalt can cause zinc that has already been deposited to redissolve. Solution purification options include cementation and chemical precipitation, whereby cementation with zinc dust is used almost without exception today. Without going into detail about leaching purification by cementation, the basic principle of cementation is the displacement of the impurity ions from the solution by the less noble zinc. During the dissolution process, zinc releases two electrons, which are absorbed by the nobler impurities in solution, which then precipitate in elementary form.

The last step is the electrolytic winning of metallic zinc, while the electrolyte gets more and more acidic. This is the reason why it is then used in the hot-acid-leaching step to dissolve pre-leached calcine and with that is neutralized again.

### (d) Lead industry

Lead is one oof the six major non-ferrous base metals, and in fourth position in terms of quantity, after aluminium, copper and zinc. While the largest producing countries, namely China, Peru and Australia, are responsible for more than 50% of primary lead production, there are also several production sites in Europe. Examples of European production sites where stockpiles are typically present as well would be KCM Plovdiv Bulgaria, Berzelius Stolberg Germany, Glencore Nordenham, Germany, Miastezko Poland, Kosovskaja Mitrovica Lead/Zinc Complex Trepca Serbia, Glencore Portovesme Italy, Glencore Britannia Refined Metals UK or also Boliden Rönnskar Sweden [46].

In addition, lead, at over 60%, has an extremely high recycling rate, which contributes significantly to annual production, especially in countries with high mobility (car/person), such as Europe. Due to the lower proportion of metallic impurities in secondary production (mainly lead acid battery recycling) compared with primary ore concentrates, and the resulting potentially interesting residues, the focus in this article should still be on primary production and the corresponding residues generated.

**Figure 4.**
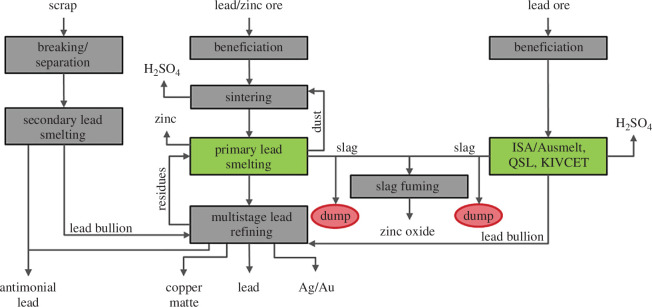
(Left) Secondary lead production route, (middle) production route via the sinter-roasting/smelting route (right) schematic flow sheet of the direct smelting route, with exemplarily named processes such as QSL, KIVCET, etc [47].

The most important lead mineral is galena, a lead sulfide (PbS). It often occurs in conjunction with sphalerite (ZnS), which is why in the past there were various co-production facilities for lead and zinc, utilizing Imperial Smelting technology/furnace (IS or ISF) or lead blast furnaces (LBF or BF). Recently, as advances in processing have made it possible to separate these polymetallic ores efficiently, these lead–zinc plants have become increasingly less important and direct smelting processes, such as Queneau–Schuhmann–Lurgi (QSL), SKS (Chinese bath smelting technology; abbreviated as SKS lead smelting process by using Chinese pinyin initials of ‘Shuikoushan’, which is the place of development), top submerged lance (TSL), and KIVCET (an acronym in Russian for oxygen flash cyclone electrothermal process) have taken over these market shares. An additional reason for the assertion of these processing technologies is owed to their improved energy utilization by combining endothermic (sinter roasting) and exothermic (smelting) process steps. As depicted in figure 4, lead production can be divided into two basic routes, namely the sintering-smelting route, either by blast furnace or Imperial Smelting Furnace, and the direct smelting route [47].

For both primary routes (ISF, BF) the sulfide minerals, after agglomeration, are converted to oxides by sintering:


$$PbS+\frac{3}{2}O_{2}↔PbO+SO_{2}.$$

As a result of possible side reactions and the formation of metallic Pb, the sinter process for lead/zinc concentrates is an inverted (air pressed from the bottom) sinter process. As a result of the strong exothermic reaction the sinter starting mixture must be kept at a certain level of sulfur, typically around 7% S by diluting with already sintered oxidic material.


$$PbS+2PbO↔3Pb+SO_{2},$$

$$PbS+2PbSO_{4}↔2Pb+2SO_{2}.$$

After the roasting, the oxidic material is reduced by carbon carriers to metallic lead. Based on the Boudouard reaction potentially formed carbon dioxide reacts with carbon further to carbon monoxide again and becomes available as a reducing agent.


PbO+C↔Pb+CO,

$$PbO+CO↔2Pb+CO_{2}.$$

Slags are inevitable wastes in pyrometallurgy, as they are a necessary by-product of purification. While in the blast furnace process, the reduction potential is kept low, to keep the zinc oxide in the slag phase, the Imperial Smelting Furnace recovers the zinc directly as a side product via the offgas, due to strong reduction potential in the reactor. While in 2004 around 55% of global primary lead was produced via the IS furnace and the blast furnace route, in 2014 it was only 30%. The reason for this can be found in the growing number of predominantly SKS productions in the Asian region and the shutdown of European plants [6,8]. Due to the migration of lead production from Europe, there are mainly dumps of residues from the lead shaft furnace and the IS route, as shown in table 4, which do not represent a complete list. Depending on the operation, approximately 500–1800 kg per tonne of produced metallic lead is generated.

**Table 4.** Amount of produced and landfilled slag from lead–zinc productions from BF (blast furnace process) and ISP (imperial smelting process).

country	location	process	in operation	slag [mio. t]	reference
France	Noyelles Godault	BF and ISP	1962–2003	~4.0	[48]
Serbia	Mitrovica	BF	1940–2010	~2.5	[49]
Macedonia	Veles	ISP	1973–2003	~1.8	[50]
Romania	Baia Mare	BF	1844–2003	~1.55	—
Romania	Copsa Mica	ISP	1965–2003	~1.5	[51]
Bulgaria	Plovdiv	BF	1963–2014	~1.1	[52]
Italy	Portovesme	ISP	1972–2004	~0.6	—
England	Avonmouth	ISP	1967–2003	~3.15	[53]
Poland	Miasteczko Slasskie	ISP	1978–today	~0.65	—

As mentioned before, new modern lead production processes combine the exothermic roasting step with the endothermic smelting and require reduction in the so-called direct smelting processes, like the QSL, KIVCET or also strongly emerging SKS technology. Typical slag compositions for the different common lead production technologies, which are still dumped to a great extent worldwide, are summarized in table 5.

**Table 5.** Slag composition of selected direct smelting processes, the imperial smelting and blast furnace process.

process	Pb (%)	Zn (%)	Fe (%)	SiO_2_ (%)	CaO (%)	reference
ISF	1.1–2.4	6.0–12.5	24.5–31.0	15.0–24.0	14.0–21.0	[54]
BF	3.6–3.8	4.8–9.0	23.7–28.8	24.8–35.0	11.5–22.0	[55,56]
QSL	5.0	15.0	20.5	21.0	16.8	[57,58]
TSL	4.0	18.0	30.0	20.0	10.0	
KIVCET	1.2–3.0	5.4–15.6	22.4–26.1	18.5–33.0	10.8–15.6	
SKS	2.0–3.0	12.0–15.0	25.0	20.0	10.0	

Accompanying elements which can also be found in the slags are typically the same metals separated during the multistage refining of crude lead, namely copper (<1.28 wt%), zinc (<7.2 wt%), silver (<120 g *t* ^−1^), and droplets of lead (<2.45 wt%), which contain traces of tin, arsenic, antimony and bismuth [46].

### (e) Spent refractory material as an indirect residue from base metals industry

Refractories are ceramic materials, which are designed to be resistant to thermal stress and to withstand physical wear and corrosion caused by chemical agents. That is why they are indispensable for high-temperature processes and consequently to pyrometallurgically produce base metals [59]. Depending on the operating requirements the refractories have to fulfil, they are produced from different materials or material combinations, usually non-metallic, such as silica, alumina, fireclay, magnesia, chromite, dolomite, carbon, silicon carbide or zirconia. In pyrometallurgical processes, the refractory materials are subject to mechanical, thermal and chemical wear mechanisms that determine their service life, ranging from a few minutes to several years [60]. Currently, the resulting vast amount of spent refractory is being landfilled to a large extent. Recycling processes have not seen a massive utilization yet, since (i) the production cost of the raw material was low, and (ii) the cost of disposal of the used refractories was not high [61]. This is adverse not only because of the inefficient use of earth’s raw materials regarding the refractory fraction, but also because infiltrated metals are not recovered.

Regarding the base metals industry, different types of spent refractories wastes and therefore different recovery potentials occur. In primary aluminium production, refractory materials, usually of the type silica–alumina, are used along with carbonaceous material for the pot lining of Hall–Héroult cells [62]. At the end of their service life, they arise as spent potlining (SPL), a hazardous waste heavily contaminated with metals, fluorides, cyanide and other compounds [63]. It can contain up to 70% graphite [64], a material that is listed as critical by the European Union [8], and arising quantities are estimated to be around 1.45 million tonnes per year. Implemented processing approaches treat around 40–50% of the generated SPL [65] but focus mainly on the use of the carbonaceous fraction as input material in other industries, while the spent refractory fraction is mostly landfilled [62]. Apart from aluminium, for the pyrometallurgical production of non-ferrous metals, refractories of the type magnesia–chrome are widely used, being viewed as the most appropriate material in terms of high-temperature stability, low thermal expansion and outstanding erosion–corrosion performance at high temperatures [60,66]. Due to the health hazard linked to chromium-containing residues, the treatment and recycling of such spent refractories have been investigated early on, but focused mainly on the recovery of the chromite fraction and disregarded the share of magnesia as well as infiltrated valuable metals [67]. Refractories from non-ferrous metallurgy can contain considerable amounts of infiltrated valuable metals. Some examples are shown in table 6.

**Table 6.** Valuable metal content of spent refractories from different metallurgical origins [wt%].

origin	Ag	Bi	Cu	Ni	Pb	Sn	Zn	reference
copper smelting	n.a.	n.a.	4.83	0.09	0.09	0.1	0.12	[68]
lead refining	0.12	2.19	0.64	n.a.	3.96	n.a.	n.a.	[69]
silver refining	1.72	3.99	0.51	0.04	5.1	n.a.	n.a.	[70]

Developing suitable recycling methods for spent refractories could lead to new secondary raw materials, both for refractory producers as well as for various metal industries while at the same time mitigating environmental hazards and supply risks.

## 4. Summary, challenges and opportunities

**Figure 5.**
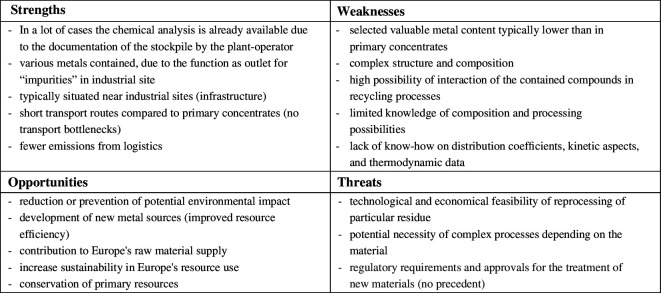
SWOT analysis of the possible processing of industrial residues from the metallurgical industry.

By going through many processing steps, the metallurgical by-products represent a complex material compared with primary ore concentrates, scrap material or in various cases also end-of-life products depending on their complexity of construction and composition. Their diversity, be it slags, dusts or even sludges, has a wide variety of morphologies and compositions and even differs from industrial plant to plant. The following figure 5 attempts to capture the possibilities of recycling these new secondary resources in a strengths, weaknesses, opportunities, threats (SWOT) analysis.

In the past, many concepts for reprocessing were investigated, but the goal was usually only the recovery of one target element or, even worse, only to avoid landfilling by using it as a building material, for example, whereby the metals contained are completely lost. In particular, the economics due to often present low concentration of a single target element are challenging and often too low for economic processing, which means that a multi-metal recovery not only takes into account the zero-waste idea but is also essential for economic efficiency. This not only makes better use of the input material, but at the same time, the accumulation of new residual materials can be minimized. The required interdisciplinary process development must be based on an in-depth characterization of the material and its contained target elements (base as well as hitch-hiker metals) to understand the behaviour of metals and trace elements in possible extraction steps and also to develop suitable strategies for influencing the behaviour of elements with the aim of extraction. This starts with a varying characterization methodology, depending on the type of residual material and its process origin. A further challenge and a reason why the field of industrial residues is a highly interesting field for the future, not only from the perspective of Europe’s security of supply with critical metals but also from a research perspective, is the fact that a basic knowledge of distribution coefficients, kinetics and of the behaviour of the elements in metallurgical extraction steps (interaction reactions) is still not available in many cases.

In this context, table 7 summarizes possible opportunities and challenges when looking at process development for these materials from the perspective of the individual residual materials: slag, dust, sludge, precipitation residues and spent refractories. Although dusts were excluded from the selected examples in this article, they would be considered here as another relevant waste stream. The table can of course only reflect a selection of relevant points and the points were selected from the authors' point of view with regard to technical and economic process development and its influence.

**Table 7.** Opportunities and challenges in the context of process development for extracting valuables from these materials.

**o**pportunities**/**/**a**dvantages	**c**hallenges
leaching residue such as the red mud (sludge)	
the structure of the concentrate is still present well-defined volume and available chemical composition can carry titanium up to 15% and hitch-hiker metals such as REE or Ga	high alkalinity very fine and moist material (thixotropy) still chemically reactive
precipitation residues such as the iron precipitate from zinc industry	
typically, high amount of material available with similar characteristics and composition well defined volume chemical analysis available	very fine, acidic and moist material low individual valuable metal content when stabilized then diluted by the addition of cement
filter dusts such as from steel mills	
existing state-of-the-art processes focus mainly on one metal, new concepts should focus on zero-waste and multi-metal recovery valuable metal content can be relatively high compared with other residues (such as slags) can carry all kinds of alloying elements (Ni, Cr, Mo, V, aside of Zn and Pb)	very fine material mixture of carryover and volatile compounds high variation of chemical composition and compounds (often not well characterized) complex morphology maybe still chemically reactive
slags such as from lead production	
stable material with known chemical composition glassy or mineral structure high amount available and easy to handle as lumpy or granulated material characterization data available	low individual valuable metal content extraction process and utilization of remains meaningful
spent refractory materials	
valuable metal content can be high but is strongly fluctuating beneficiation efforts might be easily implemented recovery of non-infiltrated refractory and metal targeted (possibility of zero-waste) rising costs for primary refractory raw materials promote economic recycling	high number of different compositions of refractory bricks additionally different infiltrations depend on the industry sector and the reactor of origin high diversity of infiltrated phases making a metallurgical extraction challenging

The potential of these materials is emphasized by the fact that these residual materials are already in Europe and are not located outside, as mine tailings are often. These by-products and residues are generated and largely deposited in European dumps and therefore represent a resource within Europe, which is therefore not subject to geopolitical risks. Historically, metallurgical production facilities have also focused primarily on one primary metal, instead of a near zero-waste production, producing a high mass of residual materials. In addition, many of the accompanying metals have only become important in the last 10–15 years due to technological advances, which means that the appropriate infrastructure for extraction still is often lacking. For this reason, too, metallurgical residues are increasingly becoming the focus of reprocessing efforts.

Especially, the development of new metal sources, reprocessing, aside of sustainable mining, such as the treatment of industrial by-products and residues, have the potential to substitute virgin raw materials, conserves primary resources and in a lot of cases also their import to Europe, linked to energy consumption and emission due to logistics. While beneficiation tailings still have the structure and morphology of the primary ores, metallurgical residues are the operations' sink for all metals that do not correspond to the target metal. As a result, unless separate extraction takes place, these accompanying metals accumulate in the associated slags, dusts and sludges of the industry and themselves become interesting concentrates, similar to polymetallic ores. This also shows similarities in the challenges of processing polymetallic concentrates, such as the targeted multi-metal extraction and the aim of developing a near-zero waste process.

The mining of the heaps not only has a positive aspect in terms of the environment but also shows a defined volume with a known composition, in contrast to new primary ore bodies, which are often only estimated on the basis of drilling samples and therefore contain a certain mining risk. Another point that applies to almost all residues from non-ferrous metallurgy is that they contain a high amount of iron and, due to the relatively low value of iron, pose a particular challenge for the possible economic utilization of these materials.

## 5. Conclusion

To avoid landfill space and to recycle the residual materials as completely as possible, multi-metal recovery and thus a zero-waste strategy should be pursued. It is of course clear that this approach depends on the degree of innovation of the treatment process and thus on its economic feasibility.

For many of the residues from the non-ferrous industry, this means thinking about the main component, iron, which contributes little to economic viability due to its availability from other sources. For this reason, iron is to be considered less in terms of the economic viability of an innovative recycling process of these residues, but rather in the context of recirculating as much as possible from the available elements and the resulting processing of the material with minimized new residual material. This underlines also the need to extract as comprehensively as possible the technologically highly important non-ferrous metals, which are often only present in trace amounts but can contribute significantly to a possible economic viability. These non-ferrous elements are also in many cases the driving force for the development of new extraction processes, as they are often classified as critical in their security of supply and so new sources are continuously sought.

This in turn requires a deep understanding of the formation processes of the residues, the associated mineralogy, structure and morphology to be able to carry out the necessary all-encompassing process development in the best possible way. This essential understanding of the above-mentioned properties of the materials and their directly linked behaviour of contained metals in metallurgical extraction steps can be achieved through an in-depth characterization of these residues.

## Data Availability

This article has no additional data.

## References

[1] Gregoir L , van Acker K . Metals for clean energy: pathways to solving Europe’s raw materials challenge. See https://eurometaux.eu/media/20ad5yza/2022-policymaker-summary-report-final.pdf (accessed 12 June 2024).

[2] European Parliament . E. C.: Regulation (EU) 2024/1252 of the European Parliament and of the Council of 11 April 2024 establishing a framework for ensuring a secure and sustainable supply of critical rawmaterials and amending Regulations (EU) No 168/2013, (EU) 2018/858, (EU) 2018/1724 and (EU)2019/1020. See https://eur-lex.europa.eu/eli/reg/2024/1252/oj (accessed 29 July 2024).

[3] European Commission . 2018 Report on critical raw materials and the circular economy. Publications Office.

[4] International resource panel . 2013 Metal recycling. Nairobi: United Nations Environment Programme.

[5] Chagnes A . 2023 Mining and processing residues. San Diego: Elsevier.

[6] Desborough GA , Smith KS , Lowers HA , Swayze GA , Hammarstrom JM , Diehl SF , Leinz RW , Driscoll RL . 2010 Mineralogical and chemical characteristics of some natural jarosites. Geochim. Cosmochim. Acta **74** , 1041–1056. (10.1016/j.gca.2009.11.006)

[7] International Energy Agency . Critical minerals market review. See https://www.iea.org/reports/critical-minerals-market-review-2023 (accessed 29 July 2024).

[8] European Commission . 2023 Study on the critical raw materials for the EU 2023: final report. Publications Office of the European Union.

[9] Werner TT , Mudd GM , Jowitt SM . 2017 The world’s by-product and critical metal resources part III: a global assessment of indium. Ore Geol. Rev. **86** , 939–956. (10.1016/j.oregeorev.2017.01.015)

[10] Moreno L . Financing and development of new mining projects. In Innovations and breakthroughs in the gold and silver industries (eds VI Lakshmanan , B Gorain ), pp. 143–156. Cham: Springer International Publishing. (10.1007/978-3-030-32549-7_7)

[11] Al-Bakri AY , Ahmed HAM , Ahmed HM , Hefni MA . 2023 Evaluation studies of the new mining projects. Open Geosci. **15** , 1–11. (10.1515/geo-2022-0466)

[12] Hoeber L , Steinlechner S . 2021 A comprehensive review of processing strategies for iron precipitation residues from zinc hydrometallurgy. Cleaner Eng. Technol. **4** , 100214. (10.1016/j.clet.2021.100214)

[13] Azimi G *et al* . 2020 Rare metal technology 2020. Cham: Springer International Publishing.

[14] Grohol M *et al* . 2023 study on critical raw materials at EU level: final report. Publications Office of the European Union. (10.2873/725585)

[15] Akcil A , Akhmadiyeva N , Abdulvaliyev R , Meshram P . 2018 Overview on extraction and separation of rare earth elements from red mud: focus on scandium. Min. Process. Extract. Metall. Rev. **39** , 145–151. (10.1080/08827508.2017.1288116)

[16] Akcil A , Swami KR , Gardas RL , Hazrati E , Dembele S . 2024 Overview on hydrometallurgical recovery of rare-earth metals from red mud. Minerals **14** , 587. (10.3390/min14060587)

[17] Dutrizac JE . 2004 The behaviour of the rare earths during the precipitation of sodium, potassium and lead jarosites. Hydrometallurgy **73** , 11–30. (10.1016/j.hydromet.2003.07.009)

[18] Jha MK , Kumar V , Singh RJ . 2001 Review of hydrometallurgical recovery of zinc from industrial wastes. Resour. Conserv. Recycl. **33** , 1–22. (10.1016/S0921-3449(00)00095-1)

[19] Dunster A . Use of ferro-silicate slag from zinc production as bound aggregate in construction. Int. Symp. Adv. Waste Manage. Recycl. 517–526.

[20] Raahauge B. E. , Williams F. S (eds). 2022 Smelter grade alumina from bauxite. Cham: Springer International Publishing; Imprint Springer. (10.1007/978-3-030-88586-1)

[21] Borra CR , Pontikes Y , Binnemans K , Van Gerven T . 2015 Leaching of rare earths from bauxite residue (red mud). Min. Eng. **76** , 20–27. (10.1016/j.mineng.2015.01.005)

[22] Sutar H . 2014 Progress of red mud utilization: an overview. Am. Chem. Sci. J. **4** , 255–279. (10.9734/ACSJ/2014/7258)

[23] Safarian J , Kolbeinsen L . Sustainability in alumina production from bauxite. In Sustainable industrial processing summit & exhibition (eds F Kongoli , P Kumar , B Senchenko , AC Silva , C Sun , W Mingan ), pp. 75–82. Environmental Science, Materials Science. See https://api.semanticscholar.org/CorpusID:109928150.

[24] Gordon JN , Pinnock WR , Moore MM . 1996 A preliminary investigation of strength development in Jamaican red mud composites. Cem. Concr. Compos. **18** , 371–379. (10.1016/S0958-9465(96)00027-3)

[25] Suss A *et al* . Specific features of scandium behavior during sodium bicarbonate digestion of red mud. In Light metals 2018 (ed. O Martin ), pp. 165–173. Cham: Springer International Publishing. (10.1007/978-3-319-72284-9_22)

[26] Salman AD *et al* . 2021 Enhancing the recovery of rare earth elements from red mud. Chem. Eng. Technol. **44** , 1768–1774. (10.1002/ceat.202100223)

[27] Gu H , Wang N , Hargreaves JSJ . 2018 Sequential extraction of valuable trace elements from bayer process-derived waste red mud samples. J. Sustain. Metall. **4** , 147–154. (10.1007/s40831-018-0164-6)

[28] Samal S . 2021 Utilization of red mud as a source for metal ions-a review. Materials **14** , 2211. (10.3390/ma14092211) 33923091 PMC8123361

[29] Chandra S . 2010 Waste materials used in concrete manufacturing. In Building materials science series. Westwood: Noyes Publications.

[30] Deady ÉA , Mouchos E , Goodenough K , Williamson BJ , Wall F . 2016 A review of the potential for rare-earth element resources from European red muds: examples from Seydişehir, Turkey and Parnassus-Giona, Greece. Mineral. Mag. **80** , 43–61. (10.1180/minmag.2016.080.052)

[31] Hind AR , Bhargava SK , Grocott SC . 1999 The surface chemistry of bayer process solids: a review. Colloids Surf. A Physicochem. Engineer Aspects **146** , 359–374. (10.1016/S0927-7757(98)00798-5)

[32] Hudson LK *et al* . Aluminum oxide. In Ullmann’s encyclopedia of industrial chemistry (eds M Bohnet , CJ Brinker , B Cornils ), pp. 1–55. Weinheim, Germany: Wiley.

[33] IEA . The role of critical minerals in clean energy transitions. See https://www.iea.org/reports/the-role-of-critical-minerals-in-clean-energy-transitions (accessed 4 March 2024).

[34] Watari T , Nansai K , Nakajima K . 2021 Major metals demand, supply, and environmental impacts to 2100: a critical review. Resour. Conserv. Recycl. **164** , 105107. (10.1016/j.resconrec.2020.105107)

[35] Davenport WG . 2002 Extractive metallurgy of copper. In Chemical, petrochemical & process. Oxford: Pergamon Press.

[36] International Copper Study Group . World copper factbook 2023. See https://icsg.org/copper-factbook/ (accessed 4 March 2024).

[37] Ndlovu S , Simate GS , Matinde E . 2017 Waste production and utilization in the metal extraction industry. Boca Raton: Taylor & Francis, CRC Press. See https://www.taylorfrancis.com/books/9781498767309.

[38] Cusano G *et al* . 2017 Best available techniques (BAT) reference document for the non-ferrous metals industries. In EUR, scientific and technical research series. Luxembourg: Publications Office of the European Union.

[39] Tian H , Guo Z , Pan J , Zhu D , Yang C , Xue Y , Li S , Wang D . 2021 Comprehensive review on metallurgical recycling and cleaning of copper slag. Resour. Conserv. Recycl. **168** , 105366. (10.1016/j.resconrec.2020.105366)

[40] Schwerdtfeger K . 1983 F. Pawlek: Metallhüttenkunde, Walter de Gruyter, Berlin, New York 1983. 865 Seiten, Preis: DM 280,—. In Berichte der Bunsengesellschaft für physikalische Chemie, p. 1230, vol. **87** . Berlin, New York: Walter de Gruyter. (10.1002/bbpc.19830871244)

[41] Tegge G . 1993 Ullmann’s encyclopedia of industrial chemistry. Weinheim, Germany: Wiley.

[42] Sinclair R.J . 2005 The extractive metallurgy of zinc. In Spectrum series/Australasian institute of mining and metallurgy, band: 13, vol. 13. Carlton, Vic: AusIMM.

[43] Dutrizac JE , Chen TT . 2000 The behaviour of gallium during jarosite precipitation. Can. Metall. Q. **39** , 1–14. (10.1179/000844300794388949)

[44] Dutrizac JE , Chen TT , Beauchemin S . 2005 The behaviour of thallium(III) during jarosite precipitation. Hydrometallurgy **79** , 138–153. (10.1016/j.hydromet.2005.06.003)

[45] Dutrizac JE , Jambor JL . 2000 Jarosites and their application in hydrometallurgy. Rev. Miner. Geochem. **40** , 405–452. (10.2138/rmg.2000.40.8)

[46] International Lead and Zinc Study Group . ILZSG: mine and smelter database.

[47] Sinclair R. J . 2009 The extractive metallurgy of lead. In Spectrum series/Australasian institute of mining and metallurgy, band: 15. Carlton, Vic: AusIMM the Australasian inst. of mining and metallurgy.

[48] Sobanska S , Deneele D , Barbillat J , Ledésert B . 2016 Natural weathering of slags from primary Pb–Zn smelting as evidenced by Raman microspectroscopy. Appl. Geochem. **64** , 107–117. (10.1016/j.apgeochem.2015.09.011)

[49] Economic Commission for Europe . 2003 Environmental performance review - yugoslavia. In Environmental performance reviews series. United Nations, Paris.

[50] Kendrovski V . 2007 Development of remediation plans with financial requirements for elimination of industrial hotspots, skopje. EUROPEAID/123674/D/SER/MK. European Agency for Reconstruction.

[51] Paulette L , Man T , Weindorf DC , Person T . 2015 Rapid assessment of soil and contaminant variability via portable x-ray fluorescence spectroscopy: Copşa Mică, Romania. Geoderma **243–244** , 130–140. (10.1016/j.geoderma.2014.12.025)

[52] Radostina Atanassova TK . 2009 Efflorescent minerals from the metallurgical waste heaps of the KCM non-ferrous metal smelter, Plovdiv, Bulgaria. Geochem. Min. Petrol. 51–63.

[53] Morrison C , Hooper R , Lardner K . 2003 The use of ferro-silicate slag from ISF zinc production as a sand replacement in concrete. Cem. Concr. Res. **33** , 2085–2089. (10.1016/S0008-8846(03)00234-5)

[54] Bohnet M , Brinker CJ , Cornils B (eds). 2003 Ullmann’s encyclopedia of industrial chemistry. Weinheim, Germany: Wiley.

[55] Seignez N , Gauthier A , Bulteel D , Buatier M , Recourt P , Damidot D , Potdevin JL . 2007 Effect of Pb-rich and Fe-rich entities during alteration of a partially vitrified metallurgical waste. J. Hazard. Mater. **149** , 418–431. (10.1016/j.jhazmat.2007.04.007) 17499917

[56] Yin NH , Sivry Y , Guyot F , Lens PNL , van Hullebusch ED . 2016 Evaluation on chemical stability of lead blast furnace (LBF) and imperial smelting furnace (ISF) slags. J. Environ. Manage. **180** , 310–323. (10.1016/j.jenvman.2016.05.052) 27240207

[57] Wu W , Xin P , Wang J . The Latest Development of Oxygen Bottom Blowing Lead Smelting Technology (eds A Siegmund , S Alam , J Grogan , U Kerney , E Shibata ). In PbZn 2020: 9th Int. Symposium on Lead and Zinc Processing, pp. 327–336. Cham: Springer International Publishing. (10.1007/978-3-030-37070-1_29)

[58] Pfennig M (ed). 1999 Schlacken in der metallurgie. Clausthal-Zellerfeld: GDMB-informationsges.

[59] Schacht C . 2004 Refractories handbook. In Mechanical engineering, band: 178. New York: Marcel Dekker. (10.1201/9780203026328)

[60] Sarkar R . 2016 Refractory technology. Taylor & francis Inc.

[61] Spyridakos A , Alexakis DE , Vryzidis I , Tsotsolas N , Varelidis G , Kagiaras E . 2022 Waste classification of spent refractory materials to achieve sustainable development goals exploiting multiple criteria decision aiding approach. Appl. Sci. **12** , 3016. (10.3390/app12063016)

[62] Holywell G , Breault R . 2013 An overview of useful methods to treat, recover, or recycle spent potlining. JOM **65** , 1441–1451. (10.1007/s11837-013-0769-y)

[63] Hou W , Li H , Li M , Cheng B . 2021 Recycling of spent refractory materials to produce al–si master alloys via the aluminum reduction cell. J. Clean. Prod. **289** , 125162. (10.1016/j.jclepro.2020.125162)

[64] Pong TK , Adrien RJ , Besida J , O’Donnell TA , Wood DG . 2000 Spent potlining – a hazardous waste made safe. Process Saf. Environ. Prot. **78** , 204–208. (10.1205/095758200530646)

[65] International aluminium: spent potlining: an introduction. See https://international-aluminium.org/wp-content/uploads/2022/09/Spent-pot-lining-Overview-Final_Eng-1.pdf (accessed 4 March 2024).

[66] Gregurek D , Ressler A , Reiter V , Franzkowiak A , Spanring A , Prietl T . 2013 Refractory wear mechanisms in the nonferrous metal industry: testing and modeling results. JOM **65** , 1622–1630. (10.1007/s11837-013-0758-1)

[67] Fang H , Smith JD , Peaslee KD . 1999 Study of spent refractory waste recycling from metal manufacturers in Missouri. Resour. Conserv. Recycl. **25** , 111–124. (10.1016/S0921-3449(98)00059-7)

[68] Han J *et al* . 2016 Innovative methodology for comprehensive utilization of spent MgO-Cr 2 O 3 bricks: copper flotation. ACS Sustain. Chem. Eng. **4** , 5503–5510. (10.1021/acssuschemeng.6b01163)

[69] Xue K , Li W , Jiao F , Qin W , Yang C . 2021 Comprehensive recovery of valuable metals from spent magnesia–chrome refractories by ferric chloride–hydrochloride leaching. J. Sustain. Metall. **7** , 898–907. (10.1007/s40831-021-00381-z)

[70] Xue K , Han J , Jiao F , Liu W , Qin W , Cai L , Xu T . 2018 Comprehensive utilization of spent magnesia-chrome refractories with gravity separation followed by flotation. Minerals Eng. **127** , 125–133. (10.1016/j.mineng.2018.08.010)

